# Clinical features and natural history of pelvic organ prolapse in South Kivu (Eastern DRC): a cross-sectional study

**DOI:** 10.11604/pamj.2025.51.48.44340

**Published:** 2025-06-17

**Authors:** Eloge Ilunga-Mbaya, Dieudonné Sengeyi Mushengezi Amani, Prosper Lukusa Tshilobo, Raha Maroyi, Alex Mutombo Baleka, Mukanire Ntankwinja, Denis Mukwege

**Affiliations:** 1 University of Kinshasa, Department of Gynecology and Obstetrics, Faculty of Medicine, Kinshasa, Democratic Republic of Congo,; 2 University of Kinshasa, Department of Pediatrics, Center for Human Genetics Research of University Clinic of Kinshasa and KU Leuven, Kinshasa, Democratic Republic of Congo,; 3 *Université Evangélique en Afrique (UEA)*, Department of Obstetrics and Gynecology, Panzi General Referral Hospital, Bukavu, The Democratic Republic of Congo

**Keywords:** Particularity, clinic, prolapse, natural history

## Abstract

**Introduction:**

pelvic organ prolapse, a common condition, is a real public health problem in developing countries. Its anamnestic and clinical features may present particularities in these environments. The objective of this study was to assess when women present for treatment and the reasons for delay in seeking care. The authors also describe the clinical particularities of prolapse in the East of the Democratic Republic of Congo.

**Methods:**

a cross-sectional study conducted on 217 women with pelvic organ prolapse consulting for the first time. Descriptive statistics and logistic regression were performed to describe clinical features and determinants of delay in seeking care.

**Results:**

the mean age was 46.38±14.12. Women under the age of 41 represents a third of the population or 36.4%. Young people (18-30 years old) represented 16.6%. Mechanical symptoms were the main reasons for consultation (63.1%) followed by sexual disorders (21.2%). Half of the population had a lateral cystocele (50.5%) followed by medio-lateral cystocele (32.1%). The average duration of symptoms was 6.67±6.85 years. The farming profession proved to be a determinant of consultation delays with adjusted Odds ratio « aOR » 3.10, 95% Confidence interval « CI », 1.055-3.481 (aOR: 3.10, 95% CI 1.055-3.481; p=0.017) and urban residence a protective factor (aOR: 0.48, 95% CI 0.24-0.97; p=0.04).

**Conclusion:**

prolapse affects women of all ages but especially a large proportion of young people in genital activity. The clinic of pelvic organ prolapse in our environment can present particularities and the time is long between the first symptoms occurrence and the decision to consult. Delays in consulting are essentially linked to the profession of farmer and the fact of residing in town is a protective factor for late consultations.

## Introduction

Pelvic organ prolapse (POP) is a debilitating condition of women, which impairs quality of life and causes social, sexual, physical and even emotional disability [1,2]. It is a prevalent condition worldwide and it represents a public health problem in developing countries with prevalence reported to be 19.7% (range 3.4% to 56.4%) [3-5]. Its management remains a major challenge in settings with limited resources given the precariousness of healthcare services and the poverty of patients [6,7]. Despite the frequency and distress of this condition, many women in resource-limited settings are reluctant to seek care [8]. Furthermore, this condition may have epidemio-clinical particularities in different regions of the world. Due to the increasing interest in prevalence studies on POP in developing countries, there is very little information on the natural history of prolapse and the clinical particularities of this condition. This study aimed to assess when women present for treatment of POP and the reasons for this delay in seeking care and also describes clinical particularities of POP in the East of the Democratic Republic of Congo.

## Methods

**Study design and setting:** this was a cross-sectional study collecting new patients presenting for the first time to the urogynecology clinic of a tertiary hospital, Panzi hospital in the city of Bukavu in the east of the Democratic Republic of the Congo.

**Study population:** women who consulted for symptoms/complaints related to POP participated in the study. A total of 217 eligible and consenting women, aged 18 to 76 years old, were registered from January 2021 to January 2022. Excluded were pregnant women, those who had already benefited from POP treatment (surgical or conservative), had congenital urogenital anomalies, and were currently under treatment for cancer or hormonal treatment. The sample size considering a prevalence of 15% with a 95% confidence interval and a margin of error of 5% was calculated at 217.

**Data collection:** patients were asked about the discovery of the initial prolapse, the time taken for symptoms to appear or their worsening, and the factors that prompted them to seek care. The questionnaire not only contained specific choices for certain questions but also had a blank space for patients to describe in their own words the reasons which led them not to consult as soon as the first symptom worsened. The questionnaire was explained in the local language by 4^th^ year interns in gynecology and obstetrics. The patients also benefited from a complete urogynecological examination including quantification of pelvic organ prolapse (POP-Q) carried out by 2 uro -gynecologists trained in this exercise. The POP-Q measurements were carried out with an empty bladder during maximum Valsalva except for 3 of these measurements including GH, Pb and TVL. The data collected during the examination were reported on the data collection form and in the medical file.

### Definitions

**Figure 1:**
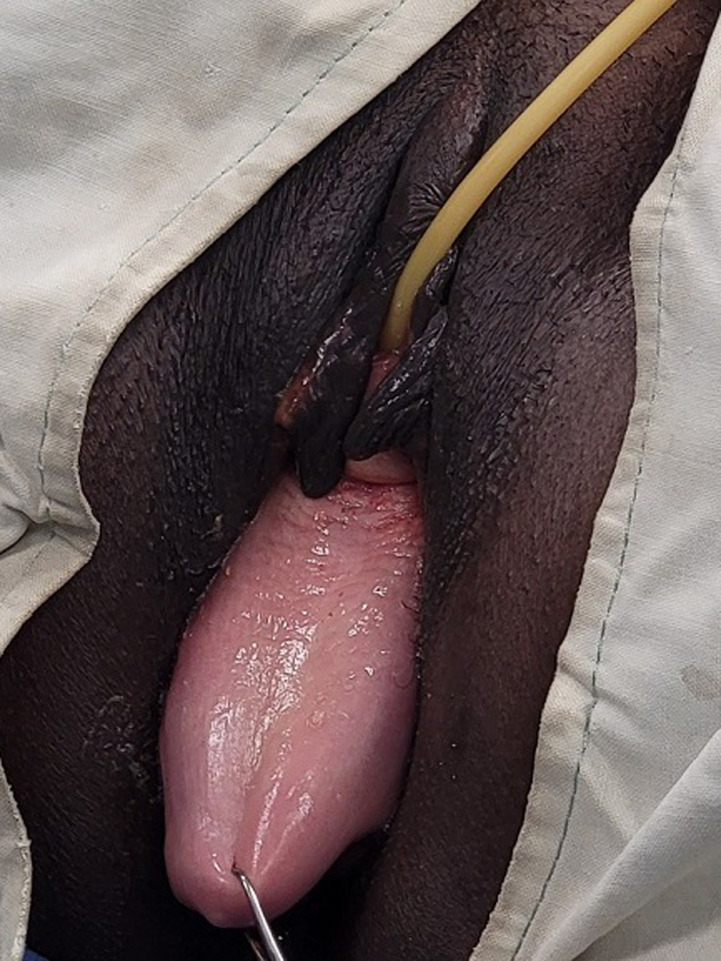
lateral cystocele + ptosis uterine

**Figure 2:**
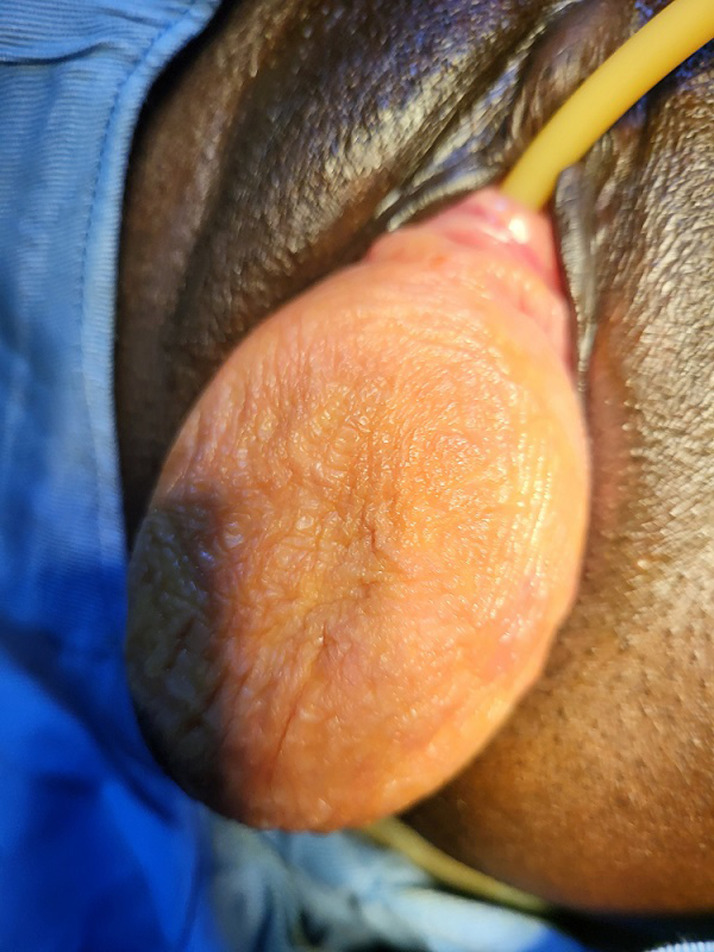
medio-lateral cystocele

The study variables were sociodemographic variables including age, profession, place of residence, clinical variables including parity (vaginal delivery only), main reason for consultation, parity at first symptom, duration of symptoms (deduced from the time of the 1^st^ symptoms or their aggravation and that of the 1^st^ consultation), the reason for the delay at the 1^st^ consultation, the types and degrees of prolapse, the compartments (levels) involved, association with the urinary incontinence. The age categories were grouped into “childbearing age” i.e. 18-30 years for the youngest and 31-40 years for the young, ''perimenopause'' and ''post-menopause '' i.e. 41-60 years and finally the ''elderly people'', i.e. over 60 years old [9]. The main reason for consultation was that reported directly by patients when the question was asked: what is the main reason that pushes you to seek for the care? Those who had never given birth were considered nulliparous and those who had 1 or 2 children were considered pauciparous. Multiparous those who had 3 to 4 children and large multiparous those who had at least 5 children. Regarding the type of cystocele, those who had a median ptosis with total disappearance of the vaginal folds making the mucosa smooth (the bladder ptosis precedes the uterus), were categorized as ''median'' therefore only a defect of the pelvic fascia. Those with ptosis of the anterior wall of the vagina with preservation of the folds of the mucosa (uterine ptosis precedes the bladder), were categorized as “lateral cystocele” (Figure 1). Those who presented a large stage 3 or 4 cystocele with complete detachment were categorized as “medio-lateral cystocele”, that is to say a mixed defect concerning the pelvic fascia and a detachment at the level of the tendinous arch of the pelvic fascia (Figure 2). Patients who consulted within the first 2 years were considered as ''early consultation'' and beyond 2 years as ''late consultation''.

**Statistical analysis:** data were recorded using Access software and analyzed with SPSS (Statistical Package for the Social Sciences) version 25. Qualitative variables were reported as proportions and quantitative variables as mean ± standard deviation. Descriptive statistics were used for basic data and differences in means were compared using chi-square tests and the student t test. Multivariable logistic regression was performed to assess the strength of association. The multivariate logistic regression models were built in a stepwise manner, following the ENTREZ method. To do this, we selected the variables that were statistically significant in the bivariate analyses. These variables were then introduced into the model, step by step, until the model was obtained. The value of P< 0.05% was considered significant.

**Ethical considerations:** each participant gave written consent and the study was approved by the national ethics committee of the School of Public Health of the University of Kinshasa under number: ESP/CE/20/2021) of 01/30/2021.

## Results

### General characteristics of the study population

A total of 217 women participated in this survey. Those who were at most 40 years old represented a proportion of 36.4%, or a third. The majority of the population came from rural areas (77.9%) and when the first symptoms appeared, 28.2% were either nulliparous (8.8%) or pauciparous (19.4%). The patients consulted mainly for mechanical symptoms ''Vulvar swelling, organ loss and feeling of heaviness'' (63.1%). The anterior compartment (cystocele) was affected in almost all cases (87.6%) and in half of the cases it was lateral cystocele (50.5%). More than half (63.1%) had stage 3 and 4 of Hysteroptosis and the 3 floors were interested in 36.9% of cases. The average duration of symptoms obtained was 6.67±6.85 years with extremes ranging from 0 to 40 years. The main reasons for delay were beliefs and ignorance (37.3%), lack of money (32.7%). Sociodemographic and clinical data of the cohort are presented in Table 1 and Table 2.

**Table 1:** socio-demographic characteristics of the study population

Variables		N	%
**Age**			
**Mean ± SD**		**46.38±14.12**	
			
	18-30	36	16.6
	31-40	43	19.8
	41-60	103	47.5
	>60	35	16.1
	Total	217	100
**Residence**			
	Rural	169	77.9
	Urban	48	21.1
	Total	217	100
**Profession**			
	No farmer	194	89.4
	Farmer	23	10.6
	Total	217	100
**Parity**			
**Mean ± SD**		**7.44±3.32**	
	Nulliparous	6	2.8
	Pauciparous	30	13.8
	Multiparous	28	12.9
	large multiparous	153	70.5
	Total	217	100
**Reason for consultation**			
	Vulvar swelling, organ loss and heaviness	137	63.1
	Urge incontinence, urinary frequency and dysuria	2	0.9
	Dyspareunia, impossibility of sexual intercourse	46	21.2
	Constipation, difficulty defecating	27	12.4
	Other (pelvic pain, abnormal discharge)	5	2.3
	Total	217	100
**Parity at first symptom**			
	Mean ± SD	5.84±3.33	
	Nulliparous	19	8.8
	Pauciparous	42	19.4
	Multiparous	47	21.7
	Large multiparous	109	50.2

**Table 2:** clinical characteristics of the population

Variables		N	%
**Type of Cystocele**			
	Median	33	17.4
	lateral	96	50.5
	Medio-lateral	61	32.1
	Total	190	100.0
**Degree of hysteroptosis**			
	1	8	3.7
	2	30	13.8
	3 - 4	137	63.1
	Total	175	80.6
**Compartments**			
	cystocele, hysteroptosis, rectocele	80	36.9
	cystocele, hysteroptosis	70	32.3
	cystocele, rectocele	40	18.4
	Hysteroptosis, Rectocele	27	12.4
	Total	217	100
**Stress urinary incontinence**			
	No	163	75.5
	Yes	53	24.5
	Total	216	100.0
**Reason for delay**			
	Lack of money	71	32.7
	Beliefs, and ignorance	81	37.3
	Shame, distance	19	8.8
	Absence of the technical platform	46	21.2
	Total	217	100.0
**Duration of symptoms**			
	**Mean ±SD**	6.67±6.85	
	0-2 years	74	34.1
	>2 -5 years	57	26.3
	>5-10 years	40	18.4
	>10 years	46	21.2
	Total	217	100

### Relationship between patient age and different variables

Table 3 shows that there are no significant difference between the stage of the disease (here considered the degree of hysteroptosis) and the age of the patients in categories (p = 0.542). Table 4shows that there is not a significant difference between the age groups and the reasons for consultation, therefore mechanical, urinary, sexual and digestive symptoms (p=0.808). Reason for delays. In bivariable analyses, women aged 40 and over (OR: 1.91, 95% CI 1.05-3.48; p=0.033) and female farmers (OR: 3.42, 95% CI 1.40-8.34; p=0.007) were identified as determinants of late consultations. Urban residence was identified as a protective factor for late consultations (OR: 0.51, 95% CI 0.26-0.99; p=0.045). In multivariable analyses, only farming as a determinant of late consultations (aOR: 3.10, 95% CI 1.055-3.481; p=0.017) and urban residence as a protective factor (aOR: 0.48, 95% CI 0.24-0.97; p=0.04) (Table 5).

**Table 3:** relationship between stage of disease and age in category

			hysteroptosis degree		
**Age in category**	**1**	**2**	**3**	**4**	**total**
	**n(%)**	**n(%)**	**n(%)**	**n(%)**	**n(%)**
**18 - 30 ans**	2(25)	2(6.7)	20(18.9)	3(9.7)	27(15.4)
**31 - 40 ans**	3(37.5)	6(20)	17(16.0)	5(16.1)	31(17.7)
**41 - 60 ans**	3(37.5)	16(53.3)	48(45.3)	17(54.8)	84(48)
**> 60 ans**	0(0)	6(20)	21(19.8)	6(19.4)	33(18.9)
**total**	8(100)	30(100)	106(100)	31(100)	175(100)

p= 0.542

**Table 4:** relationship between age group and reason for consultation

			Hysteroptosis degree			
**Age in category**	**Vulvar swelling, organ loss and heaviness**	**Dyspareunia, impossibility of sexual intercourse**	**Constipation, difficulty defecating**	**Urge incontinence, urinary frequency and dysuria**	**Other (pelvic pain, abnormal discharge)**	**Total**
	**n(%)**	**n(%)**	**n(%)**	**n(%)**	**n(%)**	**n(%)**
**18 - 30 yrs**	33(17.2)	0(0)	0(0)	1(50)	1(20)	35(16.4)
**31 - 40 yrs**	38(19.8)	3(23.1)	1(50)	0(0)	1(20)	43(20.1)
**41 - 60 yrss**	88(45.8)	8(61.5)	1(50)	1(50)	3(60)	101(47.2)
**> 60 yrs**	33(17.2)	2(15.4)	0(0)	0(0)	0(0)	35(16.4)
**Total**	192(100)	13(100)	2(100)	2(100)	5(100)	214(100)


p=0.808

**Table 5:** bivariable and multivariable logistic regression of delay determinants

Variables	Categories		Consultation	Bivariate analyze		Multivariate analyze	
		**Early n(%)**	**Late n(%)**	**COR and IC 95%**	**p**	**AOR and IC 95%**	**p**
**Age**	< 40 ans	30(40.5)	37(26.2)	1			
	≥ 40 ans	44(59.5)	104(73.8)	1.91[1.05-3.48]	**0.033**	1.88[0.98-3.60]	0.056
							
**Profession**	No farmer	14(18.9)	9(6.4)	1			
	Farmer	60(81.1)	132(93.6)	3.42[1.40-8.34]	**0.007**	3.10[1.22-7.89]	**0.017**
							
**Residence**	Rural	52(70.3)	115(82.3)	1			
	Urban	22(29.7)	25(17.7)	0.51[0.26-0.99]	**0.045**	0.48[0.24-0.97]	**0.04**
							
**Reason for delay**							
	Lack of money	28(37.8)	43(30.5)	1			
	Beliefs, and ignirance	20(27.0)	60(42.6)	1.95[0.98-3.91]	0.059	1.91[0.93-3.92]	0.078
	Shame, distance	10(13.5)	9(6.4)	0.59[0.21-1.62]	0.304	0.61[0.21-1.75]	0.354
	Absence of the technical platform	16(21.6)	29(20.6)	1.18[0.54-2.56]	0.675	1.54[0.67-3.51]	0.309

## Discussion

This study aimed to provide information on the clinical particularities of POP in our environment as well as the natural history of this pathology, that is to say the main reasons for seeking care, the time before the first consultation and the reasons for this delay in seeking care. Although often considered a condition affecting older people, we found that POP in our environment affected many young women who were sexually active and potentially wanted to become mothers. Women under 41 years old represented a proportion of 36.4%, or a third, with 16.6% of young patients aged 18 to 30 years old. These results corroborate those of an Ethiopian series where the average age of women with POP was 42.57 years. Half of the population (50.5%) was between 12 and 44 years old [3]. We also did not note any significant difference between the stages of the disease and the age categories (p = 0.542). This relationship suggests that the stages of the disease are independent of age categories, whether young, old or older. The youngest therefore present stages of POP as serious as the older ones.

Contrary to our findings, older age is often cited as a risk factor for the development of POP, with several authors suggesting a correlation between age and pelvic floor relaxation stating that the prevalence of pelvic organ prolapse in young people is lower than in older women [9]. Nygaard [10] reported in their series a low prevalence of 1.6% of POP in younger American women (under 40 years).We could be tempted to explain this large proportion of young women with prolapse in our sample by the large multiparity observed in our environment (average parity 7.44±3.32) contrary to studies carried out in countries with a high standard of living but also try to explain it by specific risk factors found in developing countries such as home births [3,11]. But it should be emphasized that a proportion of 16.6% of the women in this series were either nulliparous or pauciparous at the time of diagnosis and aged only between 18 and 30 years. Indeed, Strohbehn K *et al*. [12] makes the link between early prolapse and congenital anomalies as well as neurological and rheumatological diseases before concluding that acquired and congenital (genetic) factors can predispose young women to early genital prolapse. Jack GS *et al*. [13] reported that the risk of POP in siblings of young women is five times higher than in the general population. Bump RC *et al*. [14] subdivided the etiopathogenic mechanisms of POP into three categories: predisposing factors (genetics, collagen synthesis), inciting factors (vaginal childbirth) and contributing factors (lifestyle). Although obstetric factors with the great multiparity in our environment could explain POP in general, but the early POP found in the youngest, nulliparous could find an explanation in the predisposing factors (genetic and nutritional) acting on the synthesis of collagen. This avenue deserves further analysis. These alterations in the synthesis of collagen may be due, in nulliparous and young women, to congenital causes and nutritional (hypovitaminosis C) [15,16]. Vitamin C contributes in the stabilization of the quaternary structure of collagen. This avenue may require further research.

The patients consulted mainly for mechanical symptoms (63.1%) followed by sexual disorders (21.2%). Mechanical and urinary disorders are the 2 main reasons for consultation found in other series which for the most part included patients with average ages around 60 years [4,17-21]. The large proportion of sexual disorders in our series could be explained by the young age of our population who are in active genital activity. We also did not note any significant difference between age and reasons for consultation (p=0.808). In a retrospective survey, Kinman CL *et al*. [21] quantifiably assessed the relationship between disease discomfort and age. They found that women in the 6^th^ and 7^th^ decades of life had the highest level of discomfort compared to younger women and were more likely to seek care. In fact, the youngest were less bothered by prolapse at the same stage of the disease. An analytical investigation should be done to assess the relationship between age and reasons for consultation. The anterior compartment (cystocele) was affected in almost all cases (87.6%) and in half of the cases it was lateral cystocele (50.5%) followed by mediolateral cystocele (32.1%). More than half (63.1%) had stage 3 and 4 of Hysteroptosis. The 3 floors were interested in 36.9% of cases. In the series by Burrows, 48.2% had stage III POP and only 3.3% had stage IV of POP [22]. Ellerkmann found in his series that stage II was the most common POP and the anterior level predominated (33%) [23].

In this study, although the population is young, we find advanced stages of the disease with at least 2 compartments involved. In addition, no significant difference was noted between the age of the patients and the stage of the disease, so the youngest can present stages as advanced as the old. The reasons for this observation must be sought in etiopathogenic studies compared in the 2 groups. The average duration of symptoms obtained was 6.67±6.85 years. We see that these results are very much the opposite of the findings made in American studies [24]. Indeed, Christina LG in a cross-sectional study noted that 48% consulted immediately upon the appearance of symptoms and 80% consulted within the first year with an average time of 4 months [24]. These differences are certainly due to the poor access to health systems in our environments. On the other hand, these results are close to those of Mulat *et al*. [3] in a cross-sectional study in an Ethiopian city where the average delay in seeking care was 7 years. In our series, in multivariable analyses, only farming as a determinant of late consultations (aOR: 3.10, 95% CI 1.055-3.481; p=0.017) and urban residence as a protective factor (aOR: 0.48, 95% CI 0.24-0.97; p=0.04). This could be explained by the fact that most of these farmers live in rural areas where access to care is difficult for several reasons and that those in urban areas tend to consult earlier because the care structures are more accessible. The limitations of this study can be summarized in its descriptive nature on certain aspects. For example, an analytical study should be conducted to determine the correlations between the age of patients as well as the reasons for consultations and the stage of the disease. This study has the advantage of opening several avenues of research on aspects of POPs.

## Conclusion

POP, this debilitating pathology, affects women of all ages but especially a large proportion of young and sexually active patients. The POP clinic in our environment may present particularities. Most of these are advanced stage prolapses (3 or 4), most of which affect the 3 levels or, if not, the anterior and middle levels. For the anterior level, cystoceles are common and are often lateral and medio-lateral cystoceles (mixed mechanism). These advanced stages of POP are certainly due to a long time between the first symptoms and the decision to consult. Delays in consulting are essentially linked to the profession of farmer and the fact of residing in town is a protective factor for late consultations. These findings deserve to be taken into account for prevention and health policies.

### What is known about this topic

- Prolapse most often affects elderly and menopausal women;
- Risk factors for pelvic organ prolapse are age, multiparity, difficult childbirth, etc;
- Younger people have less advanced stages of the disease than older people.

### What this study adds

- Pelvic organ prolapse also affects a large proportion of young women and there is no significant difference between age and stage of the disease;
- There are clinical particularities in our environment;
- Women seek care late, and farming is a determinant of this delay in consultation, living in a city protects against these delays.

## References

[1] Godfrey W, Prasanna G (2012). Pelvic organ prolapse and incontinence in developing countries: review of prevalence and risk factors. Int Urogynecol J.

[2] Vandana T, Sohier E, Lauri R (2018). Demand and capacity to integrate pelvic organ prolapse and genital fistula services in low-resource settings. Int Urogynecol J.

[3] Mulat A, Solomon MA, Kiros T, Abebaw AG (2017). Reasons for delay in decision making and reaching health facility among obstetric fistula and pelvic organ prolapse patients in Gondar University hospital, Northwest Ethiopia. BMC Women's Health.

[4] Ilhan S, Cetin K, Mesut P, Enis Ö (2017). A new operation technique for uterine prolapse: Vaginally assisted laparoscopic sacrohysteropexy. Turk J Obstet Gynecol.

[5] Farjana A, Pragya G, John O, Rakibul M (2016). Prevalence of, and risk factors for, symptomatic pelvic organ prolapse in Rural Bangladesh: a cross-sectional survey study. Int Urogynecol J.

[6] Talia F, Guy DE, Hans PD (2018). Risk factors for prolapse recurrence: systematic review and meta-analysis. Int Urogynecol J.

[7] Tineke FM, Mirjam W, Joanna I, Kirsten BK (2015). Risk factors for pelvic organ prolapse and its recurrence: a systematic review. Int Urogynecol J.

[8] Dietz V van der Vaart, van der Graaf Y, Heintz P, Schraffordt SE (2010). One-year follow-up after sacrospinous hysteropexy and vaginal hysterectomy for uterine descent: A randomized study. Int Urogynecol J.

[9] Aparna DS, Neeraj K, Sujatha SR, Lennox H (2008). The age distribution, rates, and types of surgery for pelvic organ prolapse in the USA. Int Urogynecol J.

[10] Nygaard I, Barber MD, Burgio KL, Kenton K, Meikle S, Schaffer J (2008). Prevalence of symptomatic pelvic floor disorders in US women. JAMA.

[11] Ilunga-Mbaya E, Mukwege D, De Tayrac R, Mbunga B, Maroyi R, Mukanire N (2024). Exploring risk factors of pelvic organ prolapse at eastern of Democratic Republic of Congo: a case-control study. BMC Women's Health.

[12] Strohbehn K, Jakary JA, Delancey JO (2024). Pelvic organ prolapse in young women. Obstet Gynecol.

[13] Jack GS, Nikolova G, Vilain E, Raz S, Rodríguez LV (2006). Familial tranmission of genitovaginal prolapse. Int Urogynecol J.

[14] Bump RC, Norton PA (1998). Epidemiology and natural history of pelvic floor dysfunction. Obstet Gynecol Clin North Am.

[15] Akbar NDS, Dicky RS, Nuring P (2022). The difference in collagen type 1 expression in women with and without pelvic organ prolapse: a systematic review and metaanalysis. Int Urogynecol J.

[16] Nusgens BV, Humbert P, Rougier A (2002). Stimulation of collagen biosynthesis by topically applied vitamin C. European Journal of Dermatology.

[17] Vivian WS, Melissa AC, Eric RS, Charles RR, Deborah LM (2007). Variability of current symptoms in women with pelvic organ prolapse. Int Urogynecol J.

[18] Symphorosa SC, Rachel YC, Ka WY, Lai LL, Alba W (2012). Symptoms, quality of life, and factors affecting women's treatment decisions regarding pelvic organ prolapse. Int Urogynecol J.

[19] Mouritsen L (2003). Symptoms, bother and POPQ in women referred with pelvic organ prolapse. Int Urogynecol J.

[20] Marijke C, Slieker-ten H, Annelies LG, Marinus JC (2009). The prevalence of pelvic organ prolapses symptoms and signs and their relationship with bladder and bowel disorders in a general female population. Int Urogynecol J.

[21] Kinman CL, Lemieux CA, Agrawal A, Gaskins JT, Meriwether KV, Francis SL (2017). The relationship between age and pelvic organ prolapse bother. Int Urogynecol J.

[22] Burrows Lara JM, Leslie MS, Walters M, Weber AM (2004). Pelvic Symptoms in Women with Pelvic Organ Prolapse. Obstetrics & Gynecology.

[23] Mark Ellerkmann R, Geoffrey WC, Clifford FM, Mikio AN, Kenneth L (2001). Correlation of symptoms with location and severity of pelvic organ prolapse. Am Journ Obst and gyn.

[24] Christina LG, Rebecca UM, Kindra L, Dee EF (2009). Self-perceived natural history of pelvic organ prolapse described by women presenting for treatment. Int Urogynecol J.

